# PAPASH-like disease associated with a heterozygous NLRP1 variant complicated by papulopustular folliculitis

**DOI:** 10.1016/j.jdcr.2026.06.053

**Published:** 2026-06-29

**Authors:** Abdiel J. Alicea-Negrón, Angel D. Pagan-Gonzalez, Lorna M. Torres-Rosario, José R. González-Chávez, Néstor Sánchez-Colón

**Affiliations:** a Department of Medicine, School of Medicine, University of Puerto Rico Medical Sciences Campus, San Juan, Puerto Rico; b Department of Dermatology, Ponce Health Science University, Ponce, Puerto Rico

**Keywords:** autoinflammatory syndromes, neutrophilic dermatoses, NLRP1 inflammasome, PAPASH-like syndrome, PAPASH-syndrome, PsAPASH-syndrome, pustular folliculitis

## Introduction

Autoinflammatory diseases are characterized by abnormal innate immune activation leading to recurrent sterile inflammation without circulating autoantibodies or autoreactive T cells.^1^ A key mediator is the inflammasome, an intracellular complex regulating proinflammatory cytokine release. Among its sensors, NOD-like receptor family pyrin domain-containing 1 (NLRP1) is particularly relevant in cutaneous immunity, being the most prominent inflammasome sensor in human skin. Germline NLRP1 mutations have been linked to rare monogenic autoinflammatory diseases, including multiple self-healing palmoplantar carcinoma, familial keratosis lichenoides chronica, and NLRP1-associated autoinflammation with arthritis and dyskeratosis. Inflammasome dysregulation has also been implicated in neutrophilic dermatoses, where excessive innate immune activation drives sterile inflammation and cutaneous neutrophil recruitment. Syndromes such as pyogenic arthritis, pyoderma gangrenosum, and acne, pyoderma gangrenosum, acne, and hidradenitis suppurativa (PASH), pyogenic arthritis, pyoderma gangrenosum, acne, and hidradenitis suppurativa (PAPASH), psoriatic arthritis, acne, pyoderma gangrenosum, and hidradenitis suppurativa, and pyoderma gangrenosum, acne, hidradenitis suppurativa, and spondyloarthritis represent a continuum of neutrophilic skin inflammation with variable musculoskeletal involvement,^1^^,^^2^ yet the contribution of NLRP1 variants to PAPASH-spectrum phenotypes remains poorly defined. We report an 18-year-old female with a heterozygous NLRP1 variant presenting with hidradenitis suppurativa (HS), pyoderma gangrenosum (PG), acne, and inflammatory axial disease, complicated by paradoxical folliculitis and ocular inflammation following biologic exposure — highlighting the heterogeneity and therapeutic challenges of inflammasome-driven autoinflammatory disease.

## Case presentation

An 18-year-old female with no family history of similar disease and a history of chronic low back pain, autoimmune thyroiditis, and psoriasis presented with bilateral axillary HS (Hurley stage III). After failure of multiple therapies, adalimumab was initiated but discontinued due to acneiform folliculitis and uveitis. She also developed mild inflammatory acne requiring topical and oral therapy.

**Fig 1:**
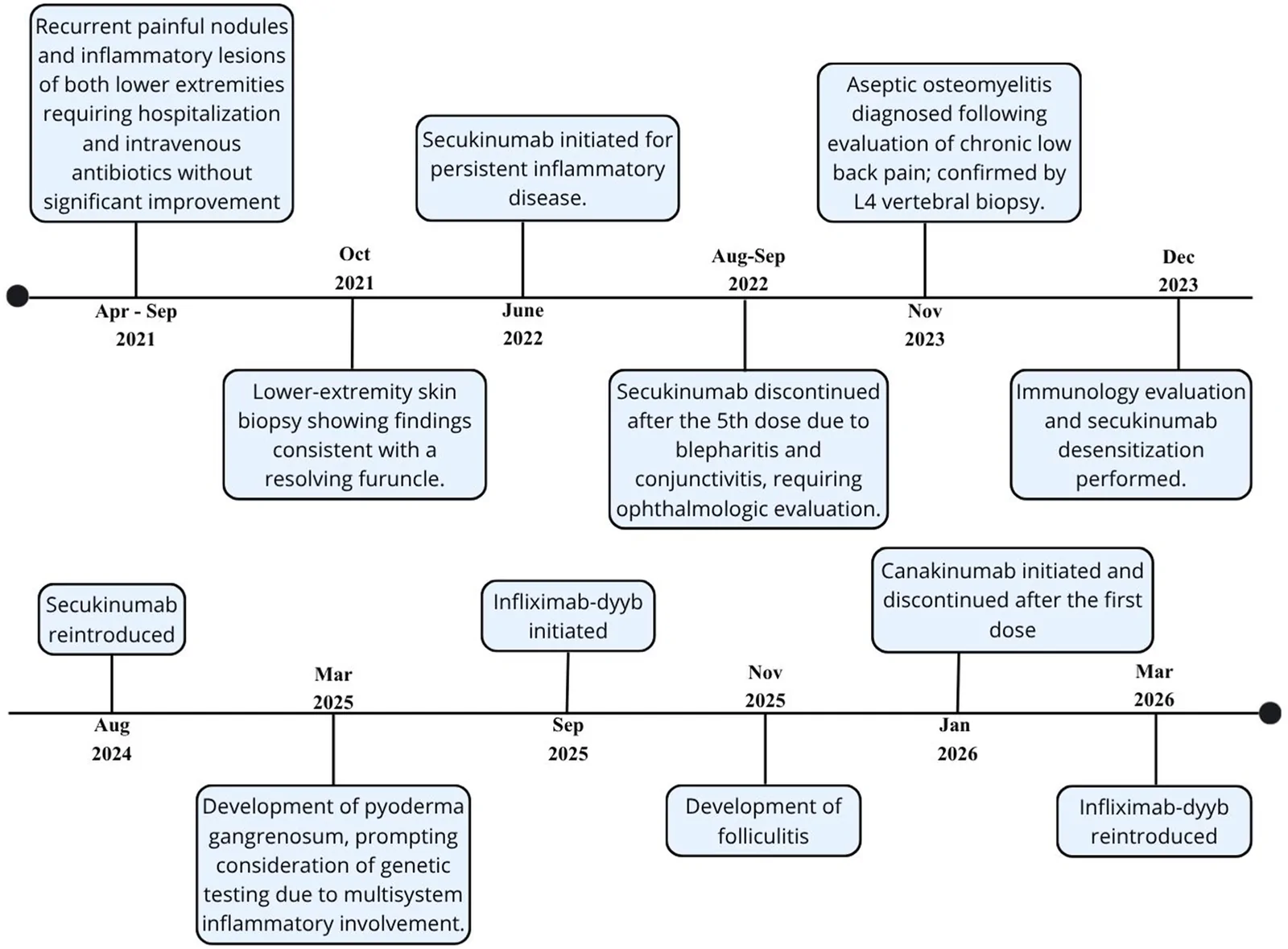
Timeline of the patient’s disease course, highlighting key clinical manifestations, treatments, and diagnostic milestones.

She subsequently developed recurrent painful nodules and inflammatory lesions of both lower extremities requiring hospitalization and intravenous antibiotics without improvement; skin biopsy was consistent with a resolving furuncle. Secukinumab was initiated for persistent HS but discontinued after the fifth dose due to blepharitis and conjunctivitis. Aseptic osteomyelitis was diagnosed and confirmed by L4 vertebral biopsy. Following immunology evaluation, secukinumab was reintroduced via subcutaneous desensitization and successfully resumed after an 8-month interval. Seven months later, she developed PG, raising suspicion for an autoinflammatory syndrome (Fig 1).

**Fig 2:**
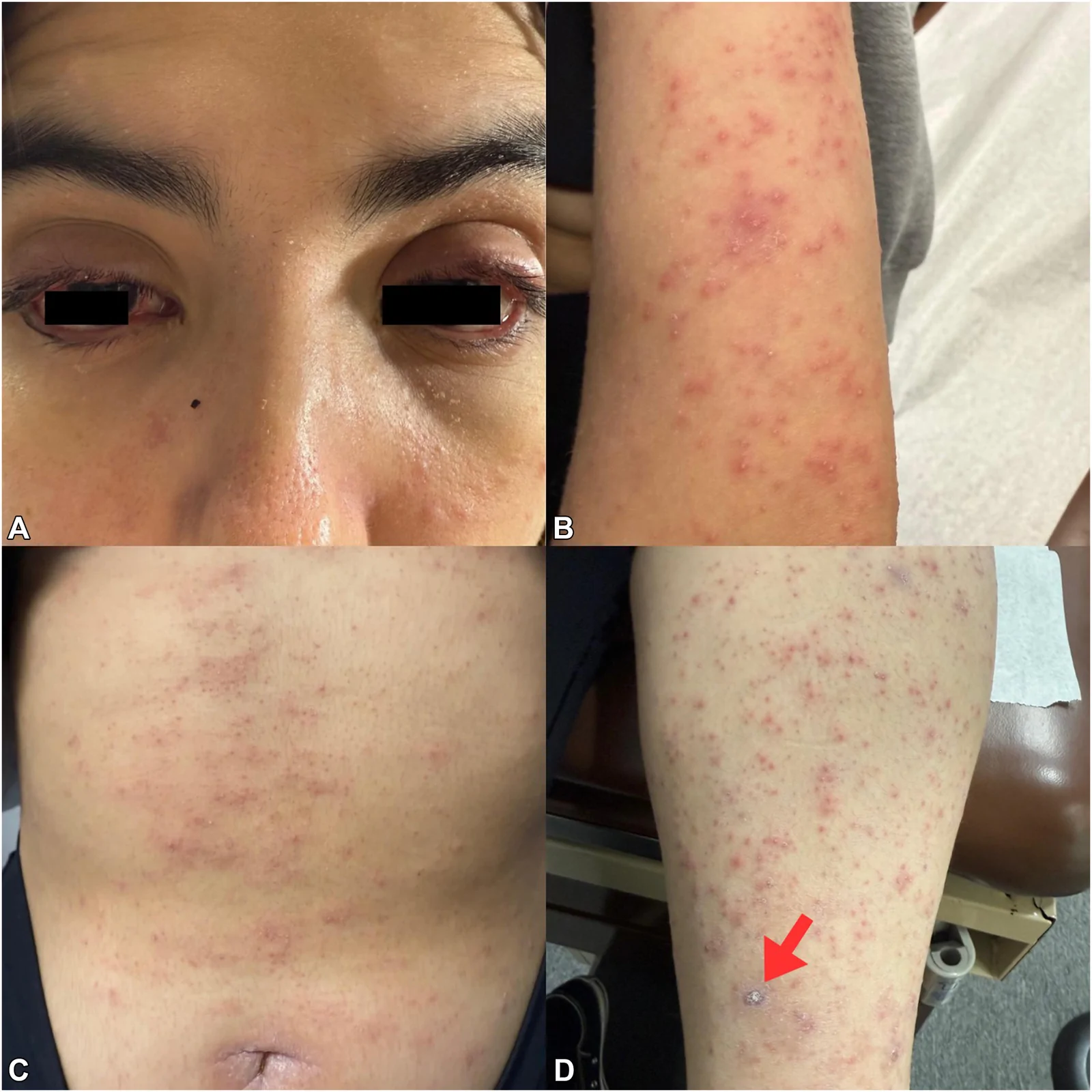
Papulopustular eruption developed after the fourth dose of infliximab. Bilateral blepharitis with periocular erythema **(A)**. Papulopustular eruption on the left arm **(B)**. Diffuse papulopustular rash on the abdomen with a well-demarcated psoriatic plaque involving the umbilicus **(C)**. Papulopustular lesions on the left thigh and the first biopsy site (*arrow*) **(D)**.

**Fig 3:**
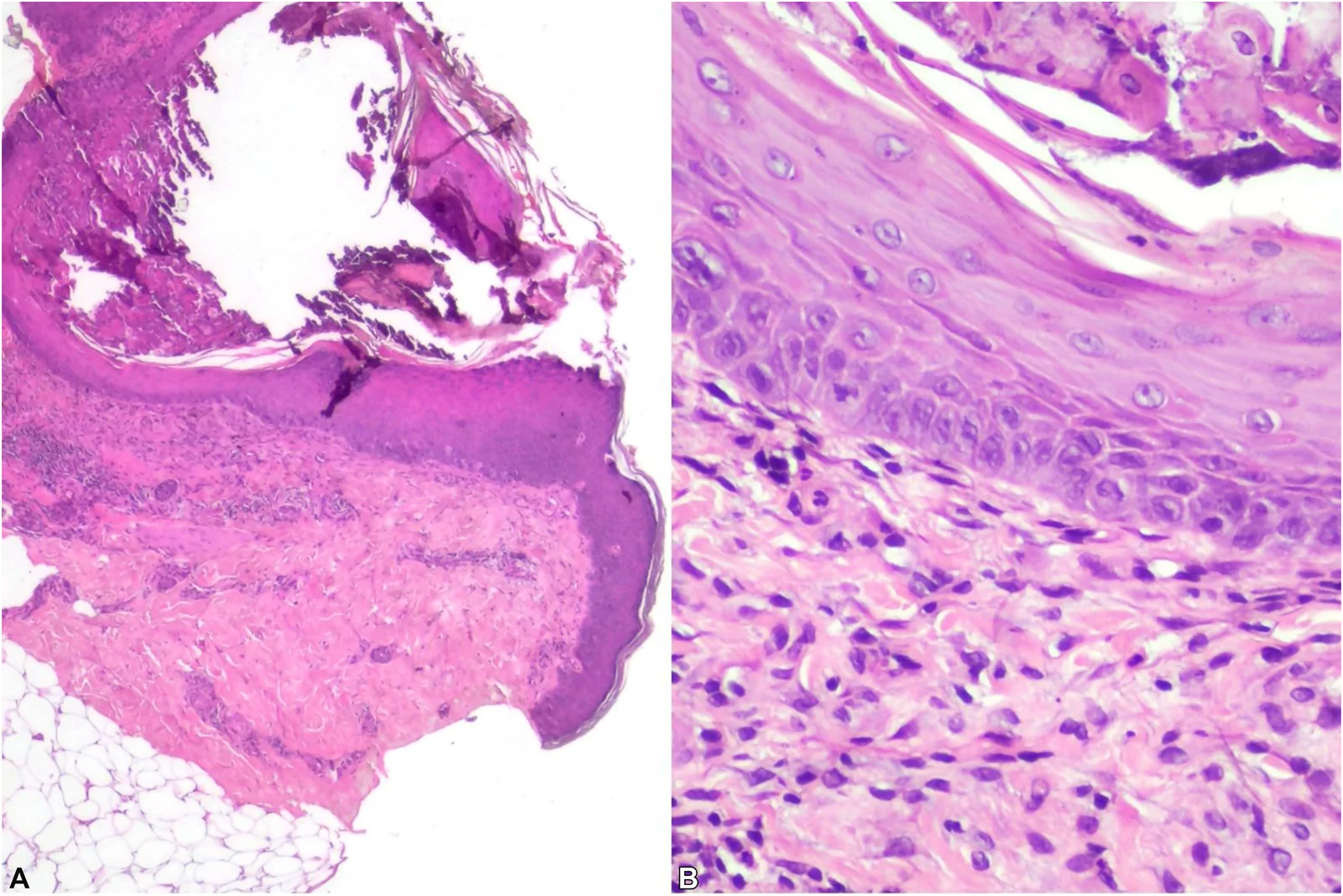
Skin biopsy from left anterior thigh pustule, showing **(A)** suppurative folliculitis with associated mixed cellular dermal inflammatory infiltrate in a superficial perivascular and perifollicular distribution (H&E; 20×). Higher magnification **(B)** demonstrates a mixed inflammatory infiltrate composed of lymphocytes, neutrophils, and scattered eosinophils involving the follicular epithelium and adjacent dermis, with associated follicular spongiosis and intraepidermal pustule formation (H&E; 100×).

Infliximab-dyyb was initiated for HS and PG, with marked improvement of skin disease and back pain. However, after the fourth infusion, she developed diffuse erythematous follicular papules and pustules (Fig 2). Skin biopsy showed a mixed infiltrate of lymphocytes, neutrophils, and scattered eosinophils (Fig 3), interpreted as neutrophil-predominant folliculitis with eosinophilic features. The eruption progressed despite infliximab discontinuation and antibiotics. Prednisone and colchicine produced near-complete clearance, though colchicine was discontinued after colitis requiring hospitalization.

**Fig 4:**
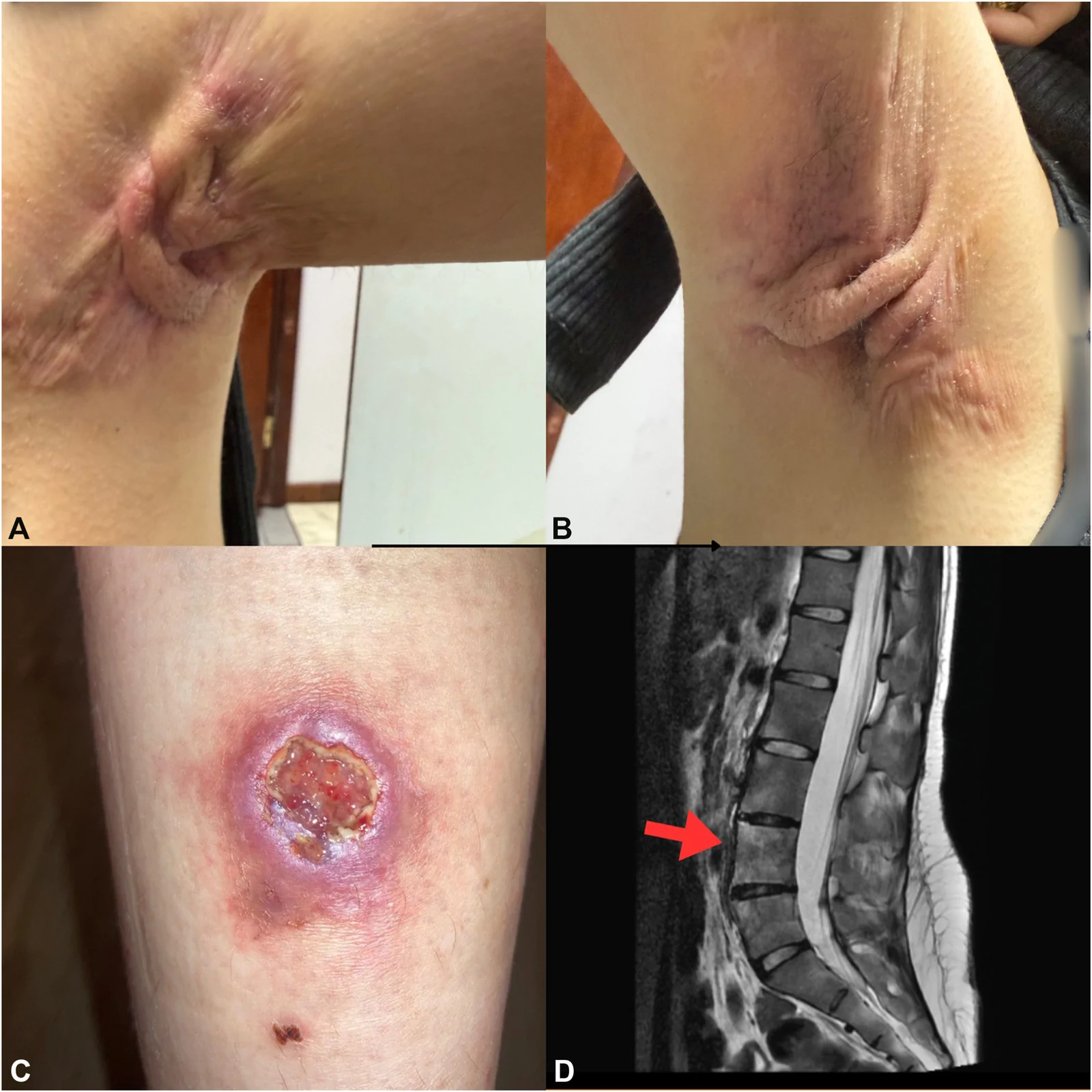
Hidradenitis suppurativa Hurley Stage III on left **(A)** and right **(B)** axilla, well-controlled, without active draining sinus tracts or cysts, 4 months after treatment with secukinumab in 2022. Pyoderma gangrenosum involving the right lower extremity in 2025 following treatment with secukinumab 300 mg subcutaneously every 2 weeks for hidradenitis suppurativa **(C)**. **D,** Sagittal lumbar MRI showing diffuse increased T2 signal with inflammatory changes at L3-L4 and L4-L5 **(D)**. The *red arrow* indicates the diffuse increased T2 signal with inflammatory changes seen on the MRI. *MRI*, Magnetic resonance imaging.

Genetic testing identified a heterozygous NLRP1 variant of uncertain significance (NM_033,004.4:c.3866G>A; p.Arg1289His); no pathogenic variants were detected in PSTPIP1, MEFV, NOD2, or NLRP3. Parental and sibling genetic testing was not performed; the patient reported no family history of similar inflammatory or dermatologic disease, and no first-degree relatives carried features consistent with this phenotype. Inflammatory markers were markedly elevated (C-reactive protein 132 mg/L; erythrocyte sedimentation rate 108 mm/h); ANA and rheumatoid factor were negative. Magnetic resonance imaging of the lumbar spine revealed partial ankylosis at L3-L4 and L4-L5 with increased T2 signal consistent with an inflammatory process (Fig 4, *D*).

Canakinumab was initiated and resulted in partial improvement of the follicular eruption; however, the patient continued to develop papulopustular lesions on the palms and soles, persistent back pain, and new PG lesions; repeat biopsy showing similar findings. Given this limited response, canakinumab was discontinued after the first dose. After a 1-month washout, infliximab was deliberately reintroduced as monotherapy without concomitant therapy, both to avoid confounding any potential adverse reaction and to assess its independent therapeutic effect. Within 1 week, the follicular eruption and PG lesions improved markedly, and back pain resolved significantly, supporting a primary therapeutic effect of tumor necrosis factor (TNF)-α inhibition. The timeline of her disease, treatments, and reactions over the past 5 years is shown in Fig 1.

## Discussion

Although our patient fulfills the core clinical components of the PAPASH spectrum — aseptic osteomyelitis, PG, acne, and HS — a strict diagnosis cannot be established given the absence of a pathogenic PSTPIP1 mutation and the nonconglobate nature of the acne. We therefore consider PAPASH-like disease or NLRP1-associated autoinflammatory disease the accurate diagnostic framework. PAPASH is classically associated with PSTPIP1 mutations, which dysregulate the pyrin inflammasome in myeloid cells, driving interleukin (IL)-1β-mediated neutrophilic inflammation. In our patient, vertebral aseptic osteomyelitis represents the osteoarticular component, distinguishing this condition from PASH syndrome.^2^

Our case harbors a gain-of-function NLRP1 mutation (c.3866G>A [p.Arg1289His]), implicating a mechanistically distinct pathway. NLRP1 is the predominant inflammasome sensor in keratinocytes, and its pathogenic mutations cause constitutive caspase-1 activation with excess IL-1β and IL-18 production, manifesting as skin-predominant autoinflammatory disease.^3^ Unlike PSTPIP1-associated autoinflammatory diseases (pyogenic arthritis, pyoderma gangrenosum, and acne; PASH; PAPASH; psoriatic arthritis, acne, pyoderma gangrenosum, and hidradenitis suppurativa; pyoderma gangrenosum, acne, hidradenitis suppurativa, and spondyloarthritis), which are driven by pyrin inflammation dysregulation, NLRP1-associated autoinflammation is keratinocyte-driven and encompasses distinct syndromes.^4^ The mutation in our patient is classified as a variant of uncertain significance, which further precludes definitive syndromic classification.

Paradoxical inflammatory reactions to adalimumab, secukinumab, and infliximab were a defining feature of this case. In the setting of a gain-of-function NLRP1 mutation, TNF-α blockade likely exacerbated the cutaneous follicular component through a cytokine seesaw effect: removal of TNF-α-mediated inhibition of plasmacytoid dendritic cells results in uncontrolled IFN-α/β production and compensatory Th1/Th17 upregulation, with IL-17A directly amplifying NLRP1 inflammasome assembly and caspase-5 activity in keratinocytes,^5^^,^^6^ paradoxically worsening the skin autoinflammatory pathway driving the papulopustular eruption. The eruption timing, persistence after drug withdrawal, histopathologic findings, and response to colchicine collectively support unmasking of a neutrophil-mediated, IL-1β-driven follicular process rather than a purely drug-induced reaction.

Importantly, this paradoxical cutaneous effect does not negate the overall therapeutic benefit of TNF-α blockade. While TNF-α inhibition exacerbates skin inflammation via IFN-α-mediated NLRP1 amplification, it simultaneously controls systemic manifestations – arthritis, PG, and HS – where TNF-α acts as the dominant downstream effector. The limited canakinumab response further illustrates this complexity: NLRP1 gain-of-function drives overproduction of IL-1β and IL-18; canakinumab neutralizes IL-1β, but persistent IL-18 sustains downstream TNF-α and IFN-γ signaling whereas Infliximab may act more broadly by disrupting upstream priming of pro–IL-1β and pro–IL-18.^7^ This treatment profile is atypical for classical PAPASH and further supports a mechanistically distinct PAPASH-like or NLRP1-associated phenotype.

This case emphasizes the importance of considering pyogenic arthritis, pyoderma gangrenosum, and acne-spectrum syndromes in patients with severe HS, PG, acne, and osteoarticular involvement refractory to standard therapies, and highlights the value of inflammasome-related genetic evaluation and personalized treatment.

## Conflicts of interest

None disclosed.
